# Potential association between M694V homozygous mutation in familial Mediterranean fever and eosinophilic intestinal inflammation: a pediatric case series

**DOI:** 10.3389/fped.2024.1419200

**Published:** 2024-08-02

**Authors:** G. Dingulu, D. Berrebi, C. Martinez-Vinson, C. Dumaine, I. Melki, J. Viala, Z. Valtuile, C. Vinit, J. P. Hugot, U. Meinzer

**Affiliations:** ^1^ General Paediatrics, Department of Infectious Disease and Internal Medicine, Robert-Debré Mother-Child University Hospital, AP-HP, Paris, France; ^2^ Reference Centre for Rheumatic, AutoImmune and Systemic Diseases in Children (RAISE), Paris, France; ^3^ General Paediatrics, Centre Hospitalier Sud Francilien, Corbeil-Essonnes, France; ^4^ Department of Pathology, Robert-Debré Mother-Child University Hospital, Assistance Publique-Hôpitaux de Paris, Paris, France; ^5^ Department of Pediatric Gastroenterology and Nutrition, Robert-Debré Mother-Child University Hospital, Assistance Publique-Hôpitaux de Paris, Paris, France; ^6^ Laboratory of Neurogenetics and Neuroinflammation, Université de Paris, Imagine Institute, INSERM, UMR, Paris, France; ^7^ Center of Clinical Investigations, INSERM CIC1426, Robert-Debré University Hospital, Assistance Publique-Hôpitaux de Paris, Paris, France; ^8^ Center for Research on Inflammation, INSERM, UMR, Université de Paris, Paris, France; ^9^ Biology and Genetics of Bacterial Cell Wall Unit, Institut Pasteur, Paris, France

**Keywords:** Familial Mediterranean Fever, microscopic colitis, iron metabolism deficiency, inflammatory bowel disease (IBD), auto-inflammatory disease

## Abstract

Familial Mediterranean fever (FMF) is the most common hereditary systemic auto-inflammatory disease. Digestive complaint is a common feature during FMF attacks. Nevertheless, digestive complaint in attack-free period has scarcely been studied. This retrospective monocentric study aimed to describe the clinical, histological, and genetic features of pediatric patients with FMF who underwent endo-colonoscopy in this setting. Out of 115 patients with a diagnosis of FMF, 10 (8, 7%) underwent endoscopy or colonoscopy. All displayed homozygote *MEFV* M694V mutation and presented chronic abdominal pain, iron deficiency, and/or growth retardation. On the histological level, all patients displayed low-grade mucosal inflammation, characterized by a moderate eosinophilic infiltrate in the lamina propria sometimes associated with increased crypt apoptosis. The proportion of patients explored with endoscopy or colonoscopy was 0.4 patients per year in our center, compared with 5.7 patients per year nationwide. This study identified a specific intestinal phenotype that does not respond to the criteria of classical inflammatory bowel disease: pediatric FMF pediatric patients with homozygous *MEFV* M694V, abdominal pain, iron deficiency, and growth retardation should benefit from specialized gastroenterological advice.

## What is known

Patients with familial Mediterranean fever (FMF) have an increased risk for developing inflammatory bowel disease (IBD). Patients with FMF also display digestive manifestations with no identified phenotype, histological pattern, or *MEFV* genotype, which does not match IBD diagnostic criteria.

## What is new

The *MEFV* M694V gene mutation may be associated with a specific intestinal phenotype, characterized by mild histologic digestive eosinophilic inflammation that does not meet the criteria for classic IBD.

Pediatric patients with FMF, abdominal pain in attack-free periods, moderate growth retardation, and chronic iron deficiency should receive specialized gastroenterological advice and endoscopic assessment.

## Introduction

Familial Mediterranean fever (FMF) is the most common hereditary systemic auto-inflammatory disease. It primarily affects populations originated from East Mediterranean territory although patients are reported worldwide. It is characterized by recurrent episodes of fever, abdominal pain, arthritis, skin manifestations, and polyserositis (1). FMF is caused by gain of function mutations in the *MEFV* gene, which encodes a protein called pyrin that has regulatory functions on the innate immune system. The M694V mutation is the most common and its homozygosity predisposes to more severe disease course, resulting in more abdominal pain, arthritis, and amyloidosis (1, 2).

Acute abdominal pain is a common feature in flares. Nevertheless, some patients also experience intestinal symptoms outside of flares. Several studies have reported that patients with FMF have an increased risk for developing classical inflammatory bowel disease (IBD), such as Crohn's disease (CD) (3) or ulcerative colitis (UC) (4). In addition, several case reports and small series have reported various gastrointestinal (GI) manifestations that could not be classified as CD or UC (4). However, the histological characteristics of these patients remain to be documented.

To that end, we described the histological lesions in 10 pediatric patients with FMF with digestive manifestations outside of FMF attacks.

## Methods

For this single-center cohort study, patients were identified, based on the International Classification of Disease (ICD), by searching a rare disease registry (BAMARA) for patients with FMF and hospital databases, both covering data from 1997 to 2020. We included the following patients: (i) aged less than 18 years at initial hospitalization at the Robert-Debré University Hospital, a tertiary children's hospital in Paris, France; (ii) with diagnosis of FMF fulfilling the PRINTO/EUROFEVER classification criteria (5); and (iii) who underwent endoscopy and/or colonoscopy. All available records of patients were retrieved to identify personal history, GI clinical complaints, blood test and fecal results, *MEFV* genotype, GI imaging, and endoscopic and histological findings. The Robert-Debré University hospital is a rare disease center in pediatric rheumatology and pediatric gastroenterology. Even so, eosinophilic patients seen in our center are very rare, including the 29 patients for eosinophilic esophagus and 2 patients for eosinophilic colitis (EC).

Biopsy specimens and histologic slides were retrieved and assessed by the same pathologist (DB) with expertise in intestinal inflammation and analyzed for histologic IBD diagnosis according to the Porto IBD group criteria (6). To quantify eosinophilic inflammation, we adapted a previously published scoring system (7) using the following scores: 0 absent, 1 present within normal limits in the lamina propria, 2 increase in lamina propria [>1/high power field (hpf) in the esophagus; >5/hpf in the stomach; >15/hpf in the duodenum; >18/hpf in the ileum; >29/hpf in the cecum; >22/hpf in the transverse colon; and >14/hpf in the sigmoid colon], 3 increase in lamina propria as above with eosinophil crypt abscesses, and 4 increase in lamina propria as above with involvement of the surface epithelium. To assess crypt apoptosis, binary grading of apoptotic bodies in the crypt epithelium was defined as absent or present.

For external validity purposes, we evaluated the occurrence of endoscopic or colonoscopy investigations in FMF patients seen in pediatric hospital settings nationwide. The data were extracted from the French Medicalization of Information Systems Program (PMSI), which is a national medico-administrative database including all patients admitted to any hospital in France. End-of-stay diagnoses were identified according to the International Statistical Classification of Diseases and Related Health Problems, 10th Revision, and following a national guideline for coding of each diagnosis. All children aged less than 18 years hospitalized with FMF from 1 January 2015 to March 2024 were included. The International Statistical Classification of Diseases and Related Health Problems, 10th Revision, diagnosis code for FMF was E850, while endoscopy and colonoscopy codes were respectively HEQE002 and HHQE005. The following data were extracted for each patient: age, hospital, and date of hospitalization. For children with multiple hospital stays, we considered the first hospital admission as date for the diagnosis.

## Results

We identified 115 patients with a diagnosis of FMF. Of these, 10 (8.7%) underwent endoscopy and/or colonoscopy and were included in the study. *MEFV* sequencing, performed by Sanger analysis (*n* = 9) or next-generation sequencing (*n* = 1), showed that all patients carried a homozygous M694V genotype. The clinical symptoms of FMF attacks are listed in Table 1. All the patients were of North African ancestry. The patients’ personal histories did not reveal digestive diseases or atopy, asthma, or urticaria.

**Table 1:** Clinical, biological, endoscopic, and histological features.

	Age at FMF diagnosis	Genotype	Clinical manifestations during FMF flares	Age at onset of GI manifestations	Clinical presentation of GI manifestations	Biological markers associated with GI manifestations	Biological iron features associated with GI manifestations	Treatment of FMF and GI manifestations	Treatment at the time of endoscopic investigation	Age at endoscopy	Macroscopic findings and histology
Patient 1	6	M694V/M694V	Fever, AR, chest pain, skin rash, aphthous oral	4	Chronic diarrhea, AP, aphthous mucous, slow growth (GH)	CRP 16 mg/L FC = 86 µg/g	Iron 3 Ferritin 25 CST NA	Colchicine and regime	Regime	13	Esophagus ulceration abnormal histology
Patient 2	6	M694V/M694V	Fever, AP, AR	2	Chronic anemia, splenomegaly	CRP < 10 mg/L Hb 6.4 g/dl VGM 50	Iron 3 Ferritin 10 CST 8.6%	Colchicine and oral iron	Oral iron	6	None abnormal histology
Patient 3	4	M694V/M694V	Fever, AP, lymphadenopathy	6	Constipation, AP, slow growth (GH)	CRP < 10 mg/L	Ferritin 10 Iron NA CST 34%	Colchicine	None	8	None abnormal histology
Patient 4	3	M694V/M694V	Fever, AP, AR	16	Chronic diarrhea, AP, vomiting, slow growth	CRP NA VS 21 mm FC = 670	Iron 2.2 Ferritin 107 CST 25.8%	Colchicine	None	16	None abnormal histology
Patient 5	3	M694V/M694V	Fever, AR, lymphadenopathy	4	AP, acute dehydration	CRP < 10 mg/L VS 10 mm	Iron 2.1 Ferritin 14 CTF N (76)	Colchicine, oral iron, and anakinra	None	4	None abnormal histology
Patient 6	5	M694V/M694V	Fever, AP, myalgia, skin rash, AR	1	AP, slow growth (GH)	CRP 40 mg/L	Iron 4.1 Ferritin 93 CST NA	Colchicine and oral iron	Oral iron	3 and 15	None abnormal histology
Patient 7	3	M694V/M694V	Fever, AP, myalgia	15	AP, perianal ulcers	CRP 63 mg/L	Iron 7.7 Ferritin 108 CST 12.2%	Colchicine and anakinra	None	17	None abnormal histology
Patient 8	1	M694V/M694V	Fever, AP, myalgia	5	Diarrhea, AP, vomiting, aphthous mucous, slow growth, acute dehydration	CRP 10 mg/L FC = 570	Iron 5 Ferritin 14 CST 6.9%	Colchicine and oral iron	IV iron None	5	2 polyps antrum abnormal histology
Patient 9	4	M694V/M694V	Fever, AP, myalgia	6	Constipation, AP, slow growth (GH), no acute dehydration	CRP NA VS 65 mm Hb 7.8 g/dl VGM 53	Iron NA Ferritin 11 CST NA	Colchicine	IV iron proton pump inhibitor	15	None abnormal histology
Patient 10	2	M694V/M694V	Fever, AP, myalgia	5	AP, constipation, failure to thrive (GH), gastroesophageal reflux	CRP < 10 mg/L	Iron NA Ferritin 10 CST NA	Colchicine and pump inhibitor	Proton pump inhibitor	12	None abnormal histology

F, fever; AR, arthralgia; AP, abdominal pain; AD, acute dehydration; FC, fecal calprotectin (µg/g); NA, non-assessed; CST, transferrin saturation coefficient (%); CTF, total transferrin (%); VGM, mean globular volume; VS, erythrocyte sedimentation rate (ESR).

Iron: normal: 12–30 µmol/L; ferritin: normal: 30–300 µg/L. Failure to thrive, weight deflection, and/or height corridor change ≥2 standard deviation.

All patients, except patient 2, were diagnosed with FMF and seen in pediatric rheumatology before digestive complaints apparition. Patients 5 and 8 were referred to the pediatric gastroenterology clinic for unexplained acute dehydration associated with chronic diarrhea. All other patients presented with insidious outside-of-flares abdominal pain, sometimes accompanied by vomiting and alternating constipation and diarrhea. Patient 7 displayed episodes of macroscopic perianal ulcers, while patients 1 and 8 displayed oral ulcers apart from febrile attacks.

All patients presented a growth failure/retardation. Regarding weight gain, patients 5 and 8 showed flattening weight curves, while the other eight patients showed a steady weight curve at a low weight level, close to −2 standard deviations (SD). Of these eight patients, four patients (patients 1, 3, 4, and 6) also showed poor height growth, with a regular curve near to −2 SD. Patients 1, 3, 6, 9, and 10 received growth hormone treatment with no significant impact on the height curve.

All patients received colchicine as soon as FMF diagnosis was confirmed. Colchicine dosage ranged from 0.5 to 2.5 mg/day. Two patients also received daily injections with an interleukin-1 (IL-1) receptor antagonist that blocks the biologic activity of IL-1, for incomplete crisis control. Treatments followed until the time of endoscopy procedures are available in Table 1.

The Biological features are reported in Table 1. Blood leucocyte counts, including eosinophil, were normal. Chronic microcytic anemia was observed in two patients. Serum C reactive protein (CRP) levels during FMF attack-free periods ranged between 0 and 60 mg/L. CRP was normal in half of the patients. In patient 7, the increased CRP (60 mg/L) was associated with an anal abscess. Fecal calprotectin was raised in two out of three patients, while albumin levels were normal when available (*n* = 3).

All patients had changes in iron metabolism (Table 1). A decreased iron blood level (<10 µmol/L) was found in all the seven patients evaluated for it. The transferrin saturation coefficient level was under 20% in three of the five patients explored. The blood ferritin level was below 30 μg/L in seven out of 10 patients. In the three remaining patients, blood ferritin level was raised, while iron blood level was low. Six patients received ineffective oral supplementation prior to endoscopy/colonoscopy.

### Ultrasound and endoscopic explorations

The ultrasound of the abdomen and bowel was normal when performed (3/10 patients). Endoscopic evaluations were performed 0–9 years after the onset of gastrointestinal complaints and were normal in 8/10 patients. They revealed an esophagus ulceration in patient 1, and an antrum polyp in patient 8.

### Histological analyses

**Figure 1:**
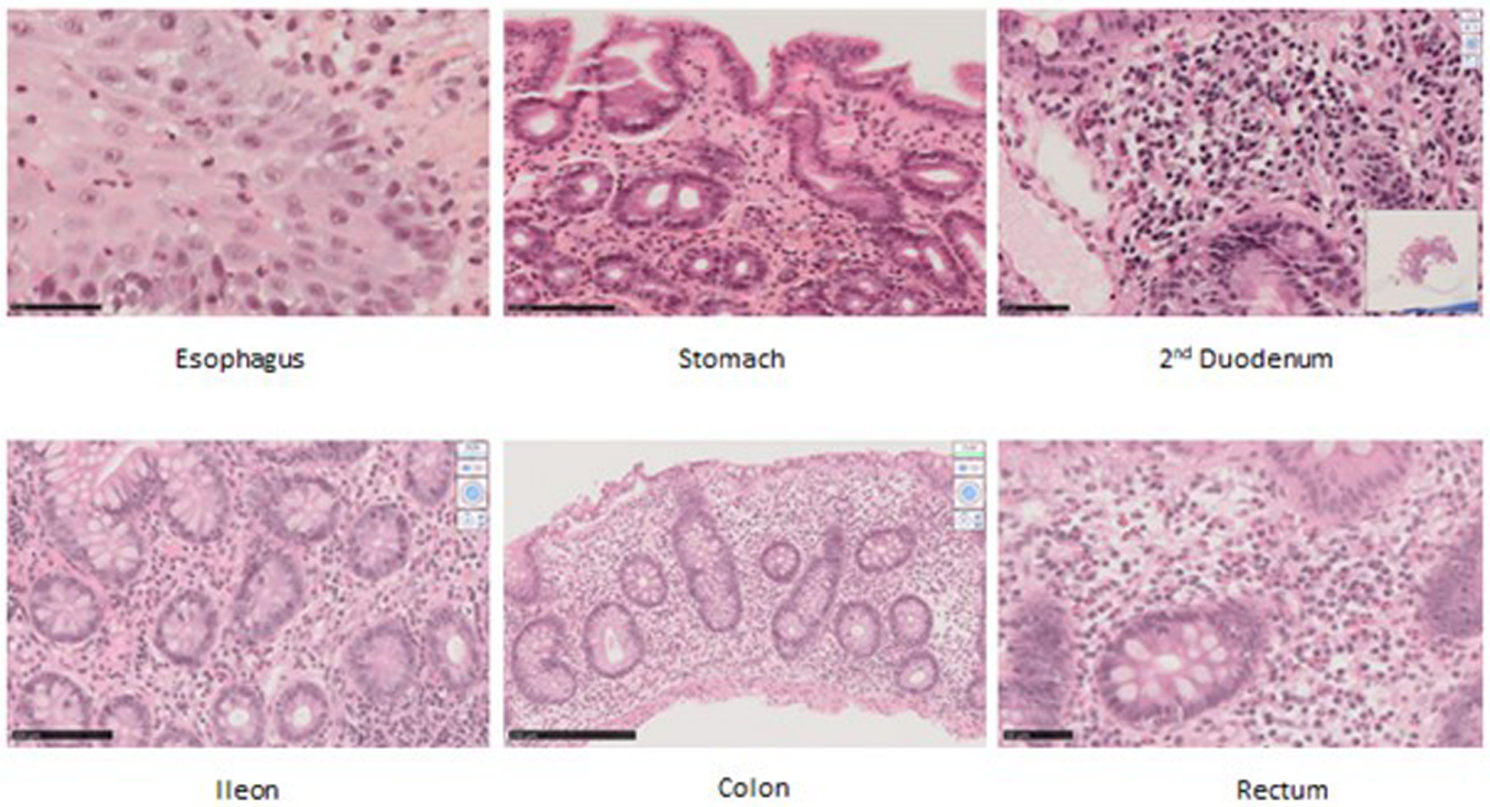
Histological samples in hematoxylin and eosin staining (HES) in original magnification (OM)*.* Patient 1’s esophagus biopsy showing eosinophilic infiltrate, score 1. OM ×40. Patient 1’s gastric biopsy showing eosinophilic infiltrate, score 2. OM ×25 ×4. Patient 8’s duodenal biopsy showing apoptosis. OM ×40. Patient 4’s ileal biopsy showing eosinophilic infiltrate, score 2. OM ×40. Patient 4’s colic biopsy showing eosinophilic infiltrate, score 2; apoptotic bodies; diastasis between bottom crypt and muscular-musculae layer. Remanence of graft versus host (GVH)-like feature. OM ×40. Patient 4’s rectal biopsy showing eosinophilic infiltrate, score 2. OM ×40.

Altogether 16 biopsies from 10 patients were available for histological analyses (Figure 1). All the biopsies had minor microscopic abnormalities with no change in the mucosal integrity and tissue organization (i.e., no crypt distortion, no gland loss). The biopsies had primarily been considered pathological in patients 7 and 8. This assessment reclassified biopsies as pathological in the other eight patients. Two microscopic abnormalities were seen: low-grade eosinophilic infiltrate (with scores ranging from 1 to 2) and crypt apoptosis. When considering data from all samples together, this pattern was observed from the stomach to the colon. Nevertheless, colonic and rectal samples were consistently pathological in all available samples, 5/5 and 2/2, respectively. Of note, crypt apoptosis was always associated with eosinophilic infiltrate. None of the patients had increased intraepithelial lymphocytes (IELs).

### Therapeutic implications

Subsequently, out of the five patients still seen in pediatric care, two were successfully maintained on anakinra and colchicine, while one remained on colchicine only, as biopsies had been considered normal. In the two patients with biopsies primarily considered pathological, intravenous (IV) iron supplementation (patients 8 and 9) showed transient efficiency. TNF-alpha blockade top-up was efficient regarding weight gain, ulcers, and inflammatory syndrome.

### External validity

**Figure 2:**
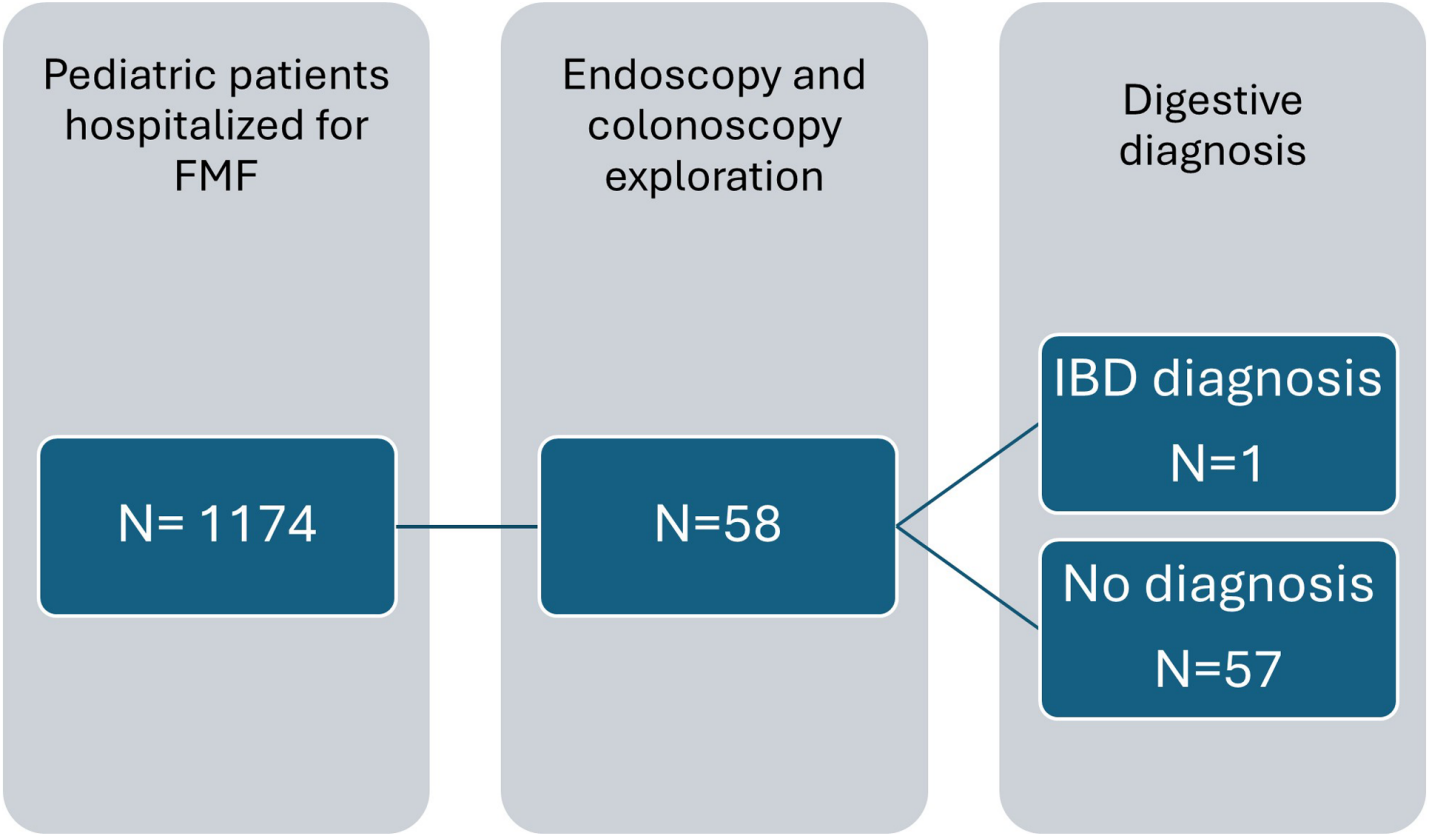
Flow chart with data from the French Medicalization of Information Systems Program from 2015 to March 2024. Description of the number of pediatric patients with FMF seen in the hospital setting, explored with endoscopy and colonoscopy.

Over a 9 year and 3 month period, out of 1,174 pediatric patients with FMF who were hospitalized, 58 patients underwent endoscopy or colonoscopy (Figure 2). Interestingly only 1 of these 58 patients was diagnosed with IBD. Therefore, 57 pediatric patients with FMF who underwent endoscopy or colonoscopy were not identified as having IBD. Hence, over the last 9 years and 3 months in France, an average of approximately six patients per year underwent endoscopy or colonoscopy for pediatric FMF treatment nationwide. In our center, we report 10 patients over a 23-year period, with an average of 0.4 patients per year.

## Discussion

Our study suggests that in children with FMF, the *MEFV* M694V homozygous gene mutation may be associated with a specific intestinal phenotype, characterized clinically by outside-of-flares abdominal pain, growth retardation, and iron deficiency and histologically by mild digestive eosinophilic inflammation found in all parts of the gastrointestinal tract with an inconsistent increase in crypt apoptosis.

Various authors have explored the digestive phenotypes associated with FMF. Early reports identified increased prevalence of classical IBD in patients with FMF (3, 8, 9). Subsequent studies performed in IBD cohorts reported increased incidence of *MEFV* gene variants in patients with IBD (4, 10). Some studies report various patients with digestive involvement with heterogeneous clinical and histological phenotype in FMF settings (11–16). Our study focused on pediatric patients with FMF matching EUROFEVER/PRINTO classification criteria followed in a French tertiary center that underwent colonoscopy (5). The 10 patients who met the inclusion criteria carried a homozygous *MEFV* M694V mutation. One patient had a complete next generation sequencing (NGS) analysis of the *MEFV* gene showing no variants other than classes 1 and 2. This finding suggests that the digestive genotype of our patient is associated with genuine homozygote M694V mutation. The genotype homogeneity in our study differs from the usual genotype prevalence in FMF cohorts (17).

A key feature was that the digestive phenotype observed in our patients relied on characteristics assessed in attack-free periods. The phenotype was mild comprising the following: (i) non-specific clinical symptoms; (ii) no or low-grade biological inflammation; and (iii) normal digestive ultrasound imaging. However, it was associated with a low growth curve and iron deficiency.

Because this histological picture does not fulfill the criteria for classic IBD, such as CD or UC, it should be analyzed with respect to microscopic colitis (MC) or EC scope (18). Since the subepithelial collagenous band and IELs were within normal range with a preserved architecture, our findings do not match diagnostic criteria for microscopic colitis. We can argue that our histological description is closer to the EC (18). Two types of EC, allergic and secondary, have been described in the literature (18). As no prior atopy was reported in our cohort, this histological phenotype should be considered as a mild form of non-allergic EC.

It is conceivable that the observed histological phenotype may be the result of colchicine digestive toxicity in patients receiving lifelong colchicine or even proton pump inhibitors (PPI). Colchicine can cause mucosal injury characterized by the crypt-villous atrophy pattern with increased mitotic rate, indicative of increased cell turnover (19). In our study, the mitotic level has not been studied, but no atrophy was observed. Our phenotype does not match the three patterns of drug-induced colitis reported (20). Moreover, the only patient in our study that underwent endoscopy before colchicine initiation did not display any difference compared with the other patients. Therefore, while we cannot exclude an influence of colchicine on the histological lesions, the observed histological presentation does not support this hypothesis. Regarding PPI, it had only been used in 2 out of the 10 patients; therefore, it can hardly be the cause of clinical symptoms and histological changes in all the patients.

Our study had some limitations. We used informatics tools that allowed for the exhaustive identification of patients with FMF who underwent endoscopy and/or colonoscopy at our hospital. Consequently, it is unlikely that any patients hospitalized for these procedures as part of FMF were missed, which limits but does not eliminate the possibility of selection bias. Furthermore, all patients examined for gastrointestinal manifestations were found to have homozygous M694V mutations and the same histological phenotype. Given that in our cohort patients with M694V homozygous mutation account for approximately 30%, i.e., 36 patients, therefore, our finding may not simply be due to coincidence or selection bias, even though we cannot formally exclude this possibility (21). The fact that all these patients have the same mutations also supports the idea of an association between this genotype and digestive signs, which does not appear to be due to chance.

Moreover, the phenotype prevalence observed in our center is not really different compared with the national data. As the PMSI data are only stored for 9 years and the ongoing year, it was not possible to retrieve older data. Nevertheless, this external validity showed that our tertiary center accounted for approximately 6% of the patients seen in France with digestive complaints leading to invasive gastrointestinal manifestations. First, it would be of great interest to confirm that the other patients match our phenotype. Second, the various therapeutic approaches and outcomes need further description and investigation.

In conclusion, patients with homozygous M694V FMF displaying abdominal pain in attack-free periods, failure to thrive, ulcers, and iron deficiency should receive specialized gastroenterological advice and endoscopic assessment. Further studies are required to identify the most appropriate treatment options and to understand whether this phenotype can also be found in adults.

## Data Availability

The original contributions presented in the study are included in the article/Supplementary Material, further inquiries can be directed to the corresponding author.
